# Sustainable corrosion inhibition of carbon steel in hydrochloric acid using repurposed expired clarithromycin as a reverse of mining

**DOI:** 10.1038/s41598-026-47188-0

**Published:** 2026-05-18

**Authors:** Mahmoud G.A. Saleh, Arej S. Al-Gorair, Hanaa M. Hawsawi, Salih S. Al-Juaid, Syed Khalid Mustafa, Metwally Abdallah, Sameer Abdullah Nooh, S. Abd El Wanees

**Affiliations:** 1 Chemistry Department, College of Science, Northern Border University, Arar, Saudi Arabia; 2 Chemistry Department, College of Science, Princess Nourah Bint Abdulrahman University, P. Box 84428, Riyadh, 11671 Saudi Arabia; 3 University College of Alwajh, University of Tabuk, Alwajh, Tabuk, Saudi Arabia; 4 Chemistry Department, Faculty of Science, King Abdulaziz University, Jeddah, 21589 Saudi Arabia; 5 Chemistry Department, Faculty of Science, University of Tabuk, Tabuk, Saudi Arabia; 6 Chemistry Department, Faculty of Science, Benha University, Benha, 13518 Egypt; 7 Departement of Information Systems, Faculty of Computing and Information Technology, King Abdulaziz University, Jeddah, 21589 Saudi Arabia; 8 Chemistry Department, Faculty of Science, Zagazig University, Zagazig, Egypt

**Keywords:** Corrosion Inhibition, Clarithromycin, Hydrogen evolution, Impedance, Gravimetry, Potentiodynamic polarization, Inhibition, Mining, Chemistry, Environmental sciences, Materials science

## Abstract

Corrosion of carbon steel in acidic environments represents a major challenge in many industrial operations, particularly during pickling, acidizing, mining, and cleaning processes. In this study, expired clarithromycin (CTM) was evaluated as a green corrosion inhibitor for carbon steel in 1.0 M hydrochloric acid using complementary chemical (weight loss and hydrogen evolution) and electrochemical (potentiodynamic polarization and electrochemical impedance spectroscopy) techniques. Surface morphology and composition were analyzed via scanning electron microscopy (SEM) and energy-dispersive X-ray spectroscopy (EDX). The corrosion rate decreased progressively with increasing CTM concentration, with inhibition efficiency exceeding 90% at 5 mM and 298 K. The protective effect is attributed to the adsorption of CTM molecules onto the steel surface, forming a stable and durable barrier. Thermodynamic analysis revealed negative ΔG°_ads_ values ranging from **−** 33.88 to − 37.58 kJ mol⁻**¹**, indicating spontaneous adsorption via a mixed physicochemical mechanism. A decrease in the adsorption equilibrium constant (*K*_ads_) at elevated temperatures suggested partial desorption of pre-adsorbed molecules. In contrast, high adsorption energy values confirmed strong interactions between CTM and the metal surface. These findings demonstrate the feasibility of repurposing expired pharmaceuticals as effective and environmentally friendly corrosion inhibitors. The study highlights a sustainable and cost-effective strategy for protecting carbon steel in acidic media, aligning with green chemistry principles and offering a practical approach to pharmaceutical waste valorization in corrosion inhibition technology.

## Introduction

Carbon steel is extensively employed in petroleum production, construction, and chemical processing due to its mechanical strength and low cost^1,2^. However, its performance deteriorates rapidly in acidic environments. Hydrochloric acid is routinely used in industrial pickling, acidizing, and surface treatment processes, where it promotes anodic dissolution of steel, resulting in accelerated degradation and high maintenance demands^3^.

Interestingly, the dissolution of metals under such aggressive conditions resembles hydrometallurgical extraction processes in mining. In mining, metals are intentionally dissolved from ores using strong chemical reagents for recovery, whereas corrosion represents an uncontrolled and undesirable metal loss under similar chemical conditions. From this perspective, corrosion inhibition can be conceptually viewed as the reverse of mining: instead of promoting metal dissolution, chemical strategies are employed to suppress it. Developing effective corrosion inhibitors is therefore essential to control electrochemical reactions at the metal/solution interface, reduce metal loss, and extend the service life of engineering materials. Repurposing expired pharmaceutical compounds as corrosion inhibitors provides a sustainable approach to controlling metal dissolution while simultaneously addressing environmental concerns associated with pharmaceutical waste.

Organic corrosion inhibitors are commonly applied to control acid-induced steel dissolution by forming protective interfacial layers. Their efficiency strongly depends on molecular features that favor adsorption, including heteroatoms (N, O, S), π-electron systems, and polar functional groups^4–12^. Azomethine (C = N) groups, for example, enhance adsorption strength due to the high electron density of the imine functionality^13^. Nitrogen- and sulfur-containing compounds, such as hydrazones, carbothioamides, chitosan derivatives, and Schiff bases, have achieved inhibition efficiencies above 90% at low concentrations^14–18^. Protection is generally attributed to compact adsorbed films formed via electrostatic interactions and partial charge transfer with the steel surface.

Nanoparticles have also gained attention as corrosion inhibitors due to their high surface area and barrier properties. When incorporated into coatings, they form dense films that limit the penetration of corrosive species, as demonstrated in recent nanocomposite systems reported by Fan et al.^19^. In parallel, sustainable and cost-effective strategies, including the repurposing of expired pharmaceutical drugs, have emerged. Such compounds often contain heteroatoms and functional groups capable of strong adsorption onto metal surfaces, providing environmentally friendly and economical corrosion protection^20^.

Expired pharmaceutical compounds offer several advantages over newly synthesized inhibitors or unexpired drugs^21–25^. They are cost-effective, environmentally sustainable, and readily available, while their complex molecular structures provide multiple active adsorption sites. Importantly, many expired drugs retain stable functional groups capable of forming protective layers on metal surfaces, supporting long-term corrosion inhibition^20^. For instance, expired Frisium, Fluimucil, and Fluconazole have shown inhibition efficiencies exceeding 90%, attributed to the presence of heteroatoms such as N, O, and S that promote strong adsorption onto metal surfaces. These compounds form protective films whose effectiveness increases with concentration and may match or even surpass that of newly synthesized, more expensive inhibitors^26–28^. Consequently, expired pharmaceuticals represent a practical and sustainable alternative for corrosion protection, combining accessibility, chemical stability, and high inhibition performance.

Clarithromycin (CTM), a semi-synthetic macrolide antibiotic, contains nitrogen and oxygen atoms as well as multiple functional groups that facilitate adsorption onto metallic surfaces. These structural features suggest that CTM can effectively form a protective layer on carbon steel in acidic media. This study evaluates expired CTM as a green, low-cost corrosion inhibitor for carbon steel in 1.0 M HCl. Its inhibition performance was assessed using chemical methods (weight loss and hydrogen evolution), electrochemical techniques (potentiodynamic polarization and electrochemical impedance spectroscopy), and surface analyses (SEM and EDX).

Thermodynamic and electrochemical analyses indicate a chemisorption-based adsorption mechanism for CTM on the steel surface, while SEM and EDX provide complementary information on morphology and elemental composition. Future work will include X-ray photoelectron spectroscopy (XPS) to directly confirm chemical bonds and molecular orientation, as well as theoretical studies such as Density Functional Theory (DFT) and Molecular Dynamics (MD) simulations to identify active adsorption sites and electronic descriptors responsible for strong inhibition behavior.

The novelty of this work lies in repurposing expired CTM as an effective corrosion inhibitor, following a waste-to-resource approach. Results demonstrate that expired CTM retains strong adsorption capability and high inhibition efficiency, highlighting its potential for sustainable corrosion protection and environmentally responsible pharmaceutical waste valorization.

## Experimental materials

### Materials

Carbon steel specimens employed in the gravimetric and gasometric experiments were fabricated from commercially available carbon steel, whose elemental composition has been reported previously^14^. The coupons were machined to a uniform size of 7.5 × 0.4 × 2.2 cm to ensure experimental consistency. Before testing, the steel surfaces were sequentially abraded with progressively finer grades of silicon carbide abrasive paper to achieve a homogeneous, smooth surface finish^17^. After polishing, the samples were rinsed with distilled water, degreased with ethanol, dried, and stored in a moisture-free environment until use.

All reagents utilized in this investigation were of analytical grade and used without additional purification. Hydrochloric acid solutions were prepared from concentrated HCl (37%, ACS reagent grade, specific gravity 1.19, approximately 12 N) supplied by Merck, with purity exceeding 99.9%. The required acid concentrations were obtained by appropriate dilution with distilled water immediately before experimentation.

### Clarithromycin (CTM)

Clarithromycin is a semi-synthetic macrolide antibiotic widely used for the treatment of respiratory, skin, and gastrointestinal infections, including those caused by *Helicobacter pylori*. Its molecular structure contains multiple heteroatoms and functional groups capable of adsorption onto metallic surfaces, supporting its potential role as a corrosion inhibitor.

The expired CTM used in this study had a manufacturer-stated expiration date of August 2025 and was used shortly after expiration. It was stored in a tightly sealed container, protected from light and moisture, and maintained at room temperature. No visible degradation (discoloration or precipitation) was observed. Reproducibility across electrochemical, weight-loss, and gasometric methods confirmed that the chemical integrity of CTM was preserved. Comparative tests with freshly purchased CTM were not performed, but the consistent adsorption behavior and inhibition efficiencies suggest no significant post-expiration loss of activity. Table 1.

**Table 1:** The physicochemical properties of clarithromycin (CTM).

Property	Elucidation
IUPAC name	(3R,4 S,5 S,6R,7R,9R,11R,12R,13 S,14R)−4-[(2,6-dideoxy-3-C-methyl-3-O-methyl-α-L-ribo-hexopyranosyl)oxy]−14-ethyl-7,12,13-trihydroxy-3,5,7,9,11,13-hexamethyl-6-[[3,4,6-trideoxy-3-(dimethylamino)-β-D-xylo-hexopyranosyl]oxy]oxacyclotetradecane-2,10-dione
Molecular structure	
Melting point:	Approximately 217–220 °C (with decomposition)
Molecular formula	C₃₈H₆₉NO₁₃
Molar mass	~ 747.96 g·mol⁻¹
Solubility	Slightly soluble in water. Freely soluble in methanol, ethanol, acetone, and chloroform. Solubility increases in acidic media due to protonation of the amino group.
Appearance	White to off-white crystalline powder

### Gravimetric (weight loss) measurements

Gravimetric experiments were carried out by accurately weighing the prepared carbon steel specimen $\:\left({W}_{1}\right)$using an analytical balance with a precision of ± 0.0001 g. The specimens were then immersed in 300 mL of 1.0 M HCl solution, either in the absence or presence of the CTM inhibitor, for a fixed immersion period of 8 h. After the exposure period, the specimens were removed, carefully cleaned to eliminate corrosion products, rinsed, dried, and reweighed $\:\left({W}_{2}\right)$. Each experiment was repeated three times, and the reported values represent the average results.

The immersion time of 8 h was selected to ensure measurable corrosion rates while maintaining stable experimental conditions in the acidic medium. This duration was sufficient to obtain reliable mass loss values and to clearly evaluate the inhibition performance of the investigated compound. Preliminary tests also indicated that extending the immersion time did not significantly alter the inhibition trend, confirming that the 8 h exposure period provides representative and reproducible results that are consistent with the electrochemical data and the objective of the study. Future work will extend the immersion studies to longer exposure periods to assess the durability and steady-state performance of the protective film, thereby providing a more comprehensive evaluation of its practical applicability.

The corrosion rate (*r*_g_) was calculated using the mass loss relation^29^:

1 $$\:{r}_{g}=\frac{{\Delta\:}W}{At}\:$$

where ΔW = (W₁−W₂) is the mass loss, A is the exposed surface area of the specimen, and *t* is the immersion time.

The surface coverage (θ) and inhibition efficiency (*η*_g_%) were evaluated according to^29^:

2 $$\:\theta\:=1-\frac{{r}_{g}}{{r}_{g}^{\circ\:}}$$

3 $$\:{\eta\:}_{w}\left(\mathrm{\%}\right)=\left(1-\frac{{r}_{g}}{{r}_{g}^{\circ\:}}\right)\times\:100$$

where *r*_g_° and *r*_g_ denote the corrosion rates measured in uninhibited and inhibited acidic solutions, respectively.

### Gasometric measurements

Gasometric measurements were carried out using the same specimen preparation procedure adopted for the gravimetric tests. The carbon steel sample was immersed in 300 mL of the test electrolyte, and the volume of hydrogen gas evolved during corrosion was collected by water displacement in the gasometric system.

After a brief induction period, the volume of the evolved hydrogen increased with immersion time. The experimental setup and gas collection assembly have been described in earlier studies^29–32^. The corrosion rate based on hydrogen evolution (*r*_H_) was calculated from the measured hydrogen volume (*V*) at a given immersion time (*t*). The surface coverage (*θ*) and inhibition efficiency (*η*_H_%) were obtained using:

4 $$\:\theta\:=1-\frac{{r}_{H}}{{r}_{H}^{\circ\:}}$$

5 $$\:{\eta\:}_{H}\left(\mathrm{\%}\right)=\left(1-\frac{{r}_{H}}{{r}_{H}^{\circ\:}}\right)\times\:100$$

where *r*_H_° and *r*_H_ represent the hydrogen evolution rates in the absence and presence of the CTM inhibitor, respectively.

### Electrochemical measurements

Electrochemical investigations were performed to further elucidate the inhibition mechanism of the CTM compound at the metal–solution interface. Potentiodynamic polarization and electrochemical impedance spectroscopy (EIS) measurements were carried out using a conventional three-electrode electrochemical cell. Carbon steel served as the working electrode, a platinum foil was used as the counter electrode, and an Ag/AgCl electrode acted as the reference electrode.

Potentiodynamic polarization measurements were performed by sweeping the electrode potential at a scan rate of 0.2 mV s⁻¹ using a VoltaLab PGZ 301 electrochemical workstation. The corrosion current densities for the uninhibited $\:\left({I}_{\mathrm{corr}}^{\circ\:}\right)\:$and inhibited $\:\left({I}_{\mathrm{corr}}\right)\:$systems were determined by extrapolating the anodic and cathodic Tafel regions to the corrosion potential $\:\left({E}_{\mathrm{corr}}\right)$. The surface coverage $\:\left(\theta\:\right)\:$and the polarization inhibition efficiency $\:\left({\eta\:}_{p}\mathrm{\%}\right)\:$were calculated using the following Eqs^33–35^.:

6 $$\:\theta\:=1-\frac{{I}_{\mathrm{corr}}}{{I}_{\mathrm{corr}}^{\circ\:}}$$

7 $$\:{\eta\:}_{p}\left(\mathrm{\%}\right)=\left(1-\frac{{I}_{\mathrm{corr}}}{{I}_{\mathrm{corr}}^{\circ\:}}\right)\times\:100$$

### Electrochemical impedance spectroscopy (*EIS*)

*EIS* measurements were conducted at open-circuit potential over a frequency range from 100 kHz to 50 mHz using a sinusoidal perturbation of 10 mV. The obtained impedance spectra were analyzed by fitting to appropriate electrical equivalent circuit models using ZSimpWin software^36^.

The charge transfer resistance values obtained in the absence (*R*_ct_°) and presence (*R*_ct_) of the CTM inhibitor were used to calculate the surface coverage and impedance inhibition efficiency (*η*_I_%) as follows^37^:

8 $$\:\theta\:=(1-\frac{{R}_{ct}^{\circ\:}}{{R}_{ct}}\:)$$

9 $$\:{\eta\:}_{I}\left(\mathrm{\%}\right)=(1-\frac{{R}_{ct}^{\circ\:}}{{R}_{ct}}\:)\times\:100$$

The double-layer capacitance (*C*_dl_) was estimated using:

10 $$\:{C}_{\mathrm{d}\mathrm{l}}={Y}^{\circ}({\omega\:}_{\mathrm{max}}{)}^{n-1}$$

where $\:{\omega\:}_{\mathrm{max}}=2\pi\:{f}_{\mathrm{max}}$, $\:{Y}^{\circ}$is the constant phase element coefficient, and *n* represents the deviation from ideal capacitive behavior.

### Surface morphology analysis

Surface morphological changes induced by the corrosive and inhibited environments were examined using scanning electron microscopy (SEM). A freshly polished carbon steel sample was used as a reference, while additional specimens were immersed in diluted hydrochloric acid and in hydrochloric acid containing 0.005 M CTM inhibitor at 25 °C. After 8 h of exposure, the samples were withdrawn, dried, and analyzed by SEM to assess surface degradation and inhibitor-induced protection.

## Results and discussion

### Mass loss investigation

**Fig. 1:**
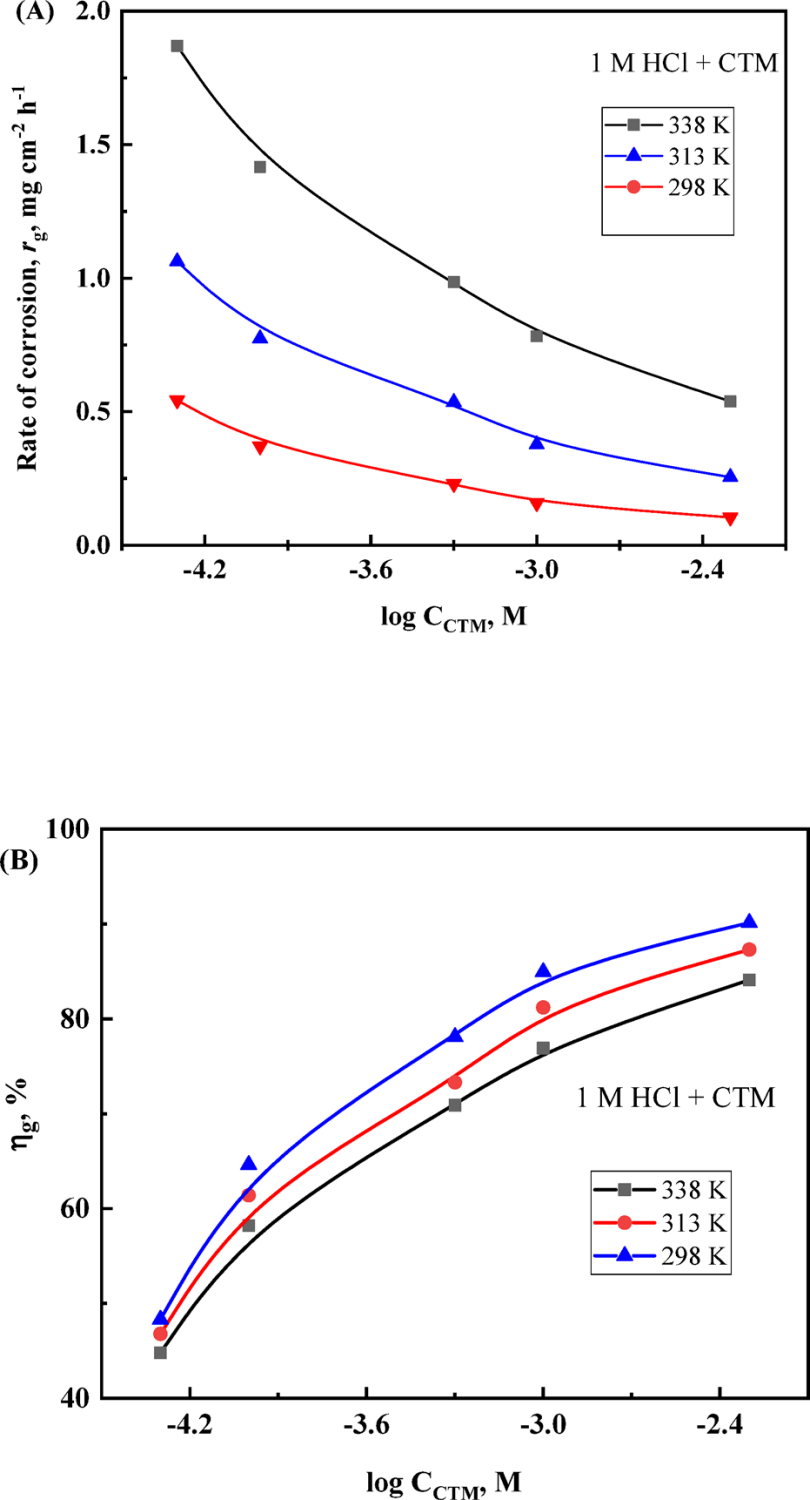
(**A**) Variation of the corrosion rate (*r*_g_) of carbon steel in 1.0 M HCl with increasing CTM concentration. (**B**) Corresponding inhibition efficiency (_w_%) plotted against the logarithm of CTM concentration.

Mass loss measurements were conducted to assess the corrosive effect of hydrochloric acid on carbon steel and to evaluate the inhibition performance of CTM at various temperatures. The inhibition efficiency (*η*_g_) is summarized in Table 2. In the absence of CTM, the corrosion rate was 1.049 mg.cm⁻².h⁻¹, decreasing to 0.543 and 0.158 mg.cm⁻².h⁻¹ upon the addition of 0.05 and 1 mM CTM, respectively, at 25 °C. Figure 1A shows a pronounced decrease in *r*_g_ values with increasing CTM concentration at all investigated temperatures.

The presence of the CTM compound in the acidic solution effectively suppressed the corrosion rate while increasing *η*_g_, as shown in Table 2; 1(A) and (B). The observed sigmoidal pattern indicates the adsorption of CTM molecules onto the carbon steel surface, which inhibits attack by chloride ions^38–40^. Slight reductions in *θ* and *η*_g_ at higher temperatures are likely due to partial desorption of adsorbed CTM into the solution, suggesting that physisorption contributes significantly to the adsorption mechanism^41^.

**Fig. 2:**
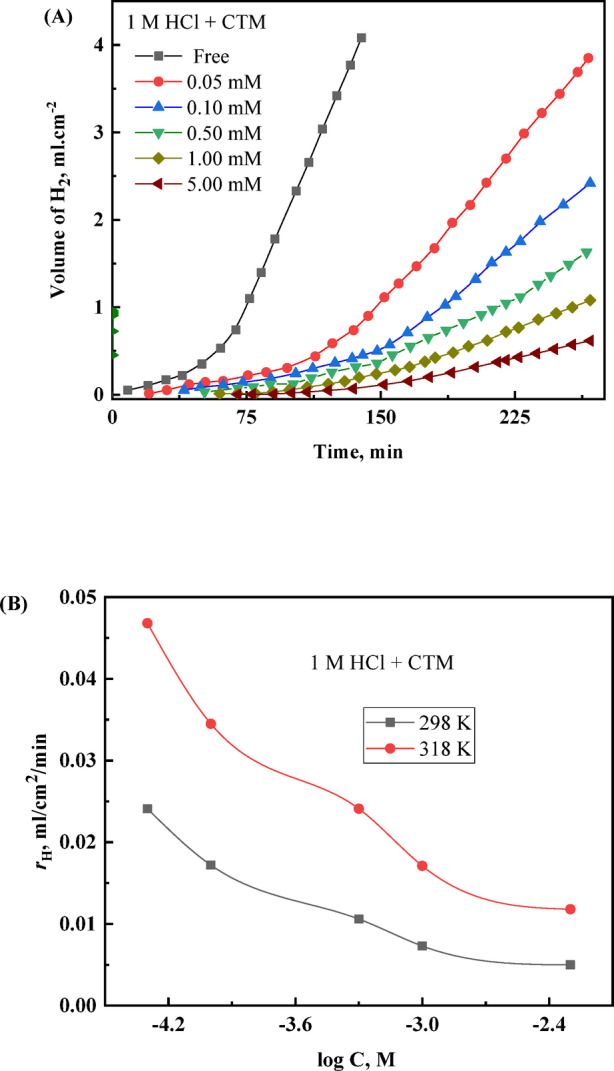
(**A**) *V–t* curves for H₂ evolution on carbon steel in 1.0 M HCl solution without and with various additions of the CTM inhibitor, and (**B**) variation of the corrosion rate (*r*_H_) as a function of log C of CTM, at 298 K.

**Table 2:** Corrosion rate (*r*_w_), surface coverage (θ), and inhibition efficiency (η_w_) of the expired CTM drug for carbon steel in 1.0 M hydrochloric acid at various temperatures (mass loss data).

Conc. mM	298 K			313 K			338 K		
	*r*_g_,mg.cm⁻².h⁻¹	θ	η_w_ (%)	*r*_g_, mg.cm⁻².h⁻¹	θ	η_w_ (%)	*r*_g_, mg.cm⁻².h⁻¹	θ	η_w_ (%)
0.00	1.0501	-	-	2.0049	-	-	3.3877	-	-
0.05	0.5430	0.48	48.33	1.0626	0.47	46.8	1.8700	0.45	44.8
0.10	0.3710	0.65	64.63	0.7739	0.61	61.4	1.4161	0.58	58.2
0.50	0.2300	0.78	78.11	0.5353	0.73	73.3	0.9858	0.71	70.9
1.00	0.1580	0.85	84.95	0.3769	0.81	81.2	0.7826	0.77	76.9
5.00	0.1040	0.90	90.13	0.2546	0.87	87.3	0.5386	0.84	84.1

### Gasometric study

The gasometric experiments were conducted to evaluate the effect of CTM on the corrosion behavior of carbon steel in 1.0 M HCl through monitoring the volume of evolved hydrogen gas (*V*_H_). The results showed that hydrogen evolution commenced after an induction period (τ) of approximately 40 min in the uninhibited acid solution. After this induction period, the hydrogen volume increased steadily with immersion time, indicating continuous dissolution of the exposed metal surface. The appearance of this induction period is attributed to the initial breakdown of the naturally formed oxide film on the steel surface by aggressive chloride ions present in the acidic medium^30,42,43^.

Upon addition of CTM, the induction period (τ) increased markedly with increasing inhibitor concentration, reaching about 113 min and 167 min for 0.01 mM and 5 mM CTM, respectively. This extension reflects the progressive adsorption of CTM molecules onto the carbon steel surface and the formation of a protective adsorbed layer that delays the initiation of corrosion. However, the value of τ decreased with increasing temperature, suggesting a partial weakening of the adsorbed inhibitor film at higher temperatures.

Figure 2A illustrates the variation of the hydrogen evolution volume (*V*_H_) with immersion time (*t*) for carbon steel in 1.0 M HCl in the absence and presence of different CTM concentrations, whereas Fig. 2B presents the corresponding hydrogen evolution rates (*r*_H_) plotted against the logarithm of CTM concentration. The obtained sigmoidal curves confirm the adsorption behavior of CTM molecules on the carbon steel surface and their significant role in suppressing metal dissolution^42^.

The hydrogen evolution rate (*r*_H_) was determined from the slope of the linear portion of the *V*_H_– *t* plot (Fig. 2A). The calculated corrosion parameters are summarized in Table 3. The results clearly show that *r*_H_ decreases significantly with increasing CTM concentration, while both the surface coverage (θ) and the inhibition efficiency (*η*_H_ %) increase. At the highest CTM concentration (5 Mm), the inhibition efficiency reaches about 90.4% at 298 K and 86.5% at 313 K. These findings demonstrate that CTM effectively retards the cathodic hydrogen evolution reaction through adsorption on the steel surface.

Furthermore, the variation of *r*_H_ with inhibitor concentration exhibits a sigmoidal trend like that observed in the gravimetric corrosion rates (*r*_g_) obtained from weight-loss measurements. This good agreement between the gasometric and gravimetric methods confirms the reliability of the obtained results and indicates that CTM effectively suppresses both the anodic dissolution of carbon steel and the cathodic H_2_ evolution reaction. The slight decrease in inhibition efficiency with increasing temperature suggests partial desorption of the inhibitor molecules from the metal surface, supporting a predominantly physisorption adsorption mechanism^44^.

**Table 3:** Corrosion rate (*r*_H_, mL.cm⁻².min⁻¹), surface coverage (θ), and protection efficiency (*η*_H_ %) for carbon steel in hydrochloric acid solution containing CTM at 298 and 313 K. (*Values in parentheses correspond to the data calculations of CTM inhibitor at 313 K*).

Conc. (mM)	*r*_H_, mL.cm⁻².min⁻¹	θ	*η*_H_
0.00	0.0477 [0.0877]^*^	---	---
0.05	0.0247 [0.0468]	0.489 [0.466]	48.3 [46.6]
0.10	0.0172 [0.0345]	0.639 [0.607]	63.9 [60.7]
0.50	0.0106 [0.0241]	0.778 [0.725]	77.8 [72.5]
1.00	0.0073 [0.0171]	0.847 [0.805]	84.7 [80.5]
5.00	0.0046 [0.0118]	0.904 [0.865]	90.4 [86.5]

### Potentiodynamic study, Pd

**Fig. 3:**
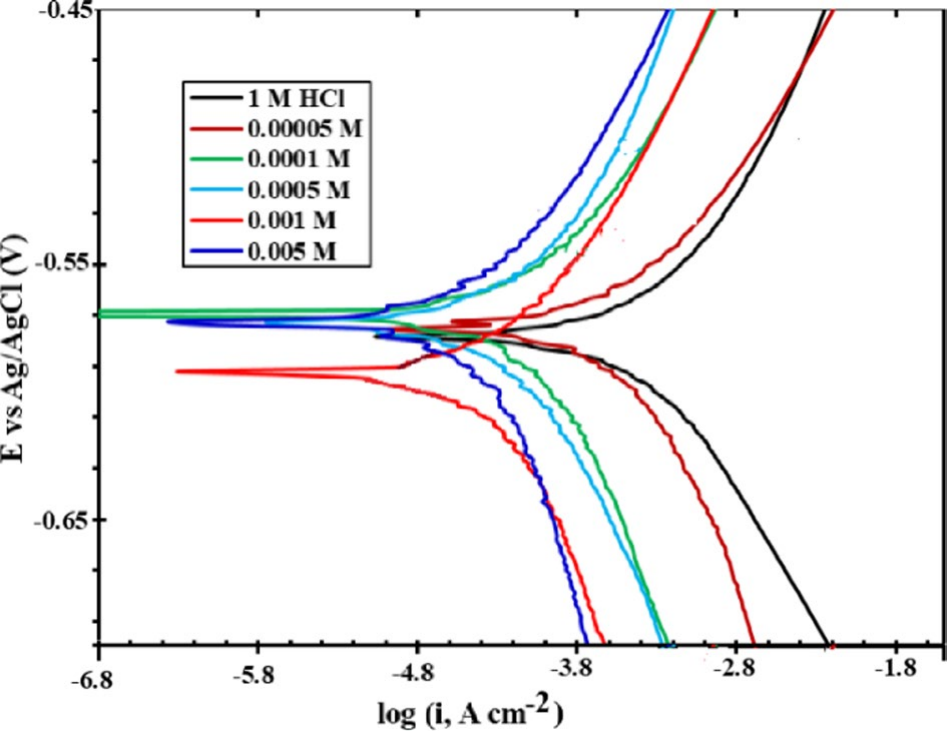
Potentiodynamic polarization curves of carbon steel in 1.0 M HCl in the absence and presence of different concentrations of the CTM inhibitor, at 25 Cᵒ.

The potentiodynamic polarization behavior of carbon steel in both corrosive and inhibited hydrochloric acid solutions is presented in Fig. 3. The electrochemical parameters, including corrosion potential (*E*_corr_), polarization resistance (*R*_p_), corrosion current density (*I*_*corr*_), anodic and cathodic Tafel slopes (*β*_a_ and *β*_c_), surface coverage (*θ*), and inhibition efficiency (*η*_p_), were extracted from the polarization curves and are summarized in Table 4.

In uninhibited 1.0 M HCl, the corrosion current density (*I*_corr_) was 1.082 mA·cm⁻². Upon addition of CTM, a significant decrease in *I*_corr_ was observed, and the values progressively declined with increasing inhibitor concentration. The corrosion current density decreased to 0.551 mA cm⁻² at 0.05 mM and further to 0.103 mA cm⁻² at 5 mM CTM. This pronounced reduction in *I*_corr_ value confirms the strong inhibitory effect of CTM on the corrosion of carbon steel in the acidic medium.

The anodic and cathodic Tafel slopes showed no significant changes upon the addition of CTM, indicating that the fundamental corrosion mechanism remains unaffected in the presence of the inhibitor^45,46^. Moreover, the polarization curves reveal that the presence of CTM does not induce a substantial shift in *E*_*corr*_ relative to the blank HCl solution. The observed displacement in corrosion potential (Δ*E*_*corr*_) was within ± 30 mV, suggesting that CTM effectively suppresses both the anodic iron dissolution and cathodic hydrogen evolution reactions. Accordingly, CTM can be classified as a mixed-type corrosion inhibitor^46–49^.

The inhibition efficiency (*η*_p_) was calculated from the corrosion current densities obtained in the absence and presence of CTM. The values of *η*_p_ increased with increasing CTM concentration, consistent with the observed decrease in *I*_corr_. This behavior confirms the effective blocking of both anodic and cathodic active sites on the carbon steel surface by adsorbed CTM molecules^17^.

The addition of CTM markedly enhanced the polarization resistance ($\:{R}_{\mathrm{p}}$) of carbon steel, increasing it from 43 Ω cm² in the blank solution (1.0 M HCl) to 165 Ω cm² and 447 Ω cm² in the presence of 0.05 mM and 5 mM CTM, respectively. This enhancement is attributed to the adsorption of CTM molecules onto the metal surface, leading to the formation of a protective film that limits corrosive attack^50^. The progressive increase in both $\:{R}_{\mathrm{p}}$and inhibition efficiency (η_p_) with rising CTM concentration indicates the development of a denser and more compact inhibitor layer, resulting in improved corrosion protection^17^.

At a CTM concentration of 5 mM, the $\:{R}_{\mathrm{p}}$value (447 Ω cm²) was higher than the charge-transfer resistance obtained from *EIS* measurements (*R*_ct_ = 242.6 Ω cm²). This discrepancy can be explained by the different electrochemical principles underlying the two techniques. While $\:{R}_{\mathrm{p}}$, derived from linear polarization measurements, represents the overall resistance to anodic and cathodic processes, *R*_ct_, obtained from *EIS*, specifically reflects the charge-transfer kinetics at the metal–solution interface. Such differences are commonly observed at elevated inhibitor concentrations, where adsorption effects become more pronounced. Under these conditions, $\:{R}_{\mathrm{p}}\:$is often considered more reliable, as it provides a frequency-dependent evaluation of interfacial electrochemical behavior.

**Table 4:** Electrochemical corrosion inhibition factors calculated for the CTM inhibitor, at 25 ᵒC.

Conc., mM	E_corr_, mV, SCE	I_corr_, mA cm^− 2^	Β_a_, mV dec^− 1^	Β_c_, mV dec^− 1^	Rp, Ω cm²	η_*p*_, %
0.00	−524	1.082	153	−179	43	-
0.05	−525	0.551	154	−177	165	49.1
0.10	−526	0.380	162	−187	195	64.9
0.50	−554	0.234	156	−168	202	78.4
1.00	−542	0.155	165	−168	281	85.8
5.00	−526	0.103	146	−151	447	90.5

Overall, the simultaneous increase in $\:{R}_{\mathrm{p}}\:$ and *η*_p_ with increasing CTM concentration confirms the progressive surface coverage by CTM molecules and supports the formation of a stable adsorbed layer that effectively isolates the metal surface from the aggressive acidic medium^17^.

### The impedance, EIS study

**Fig. 4:**
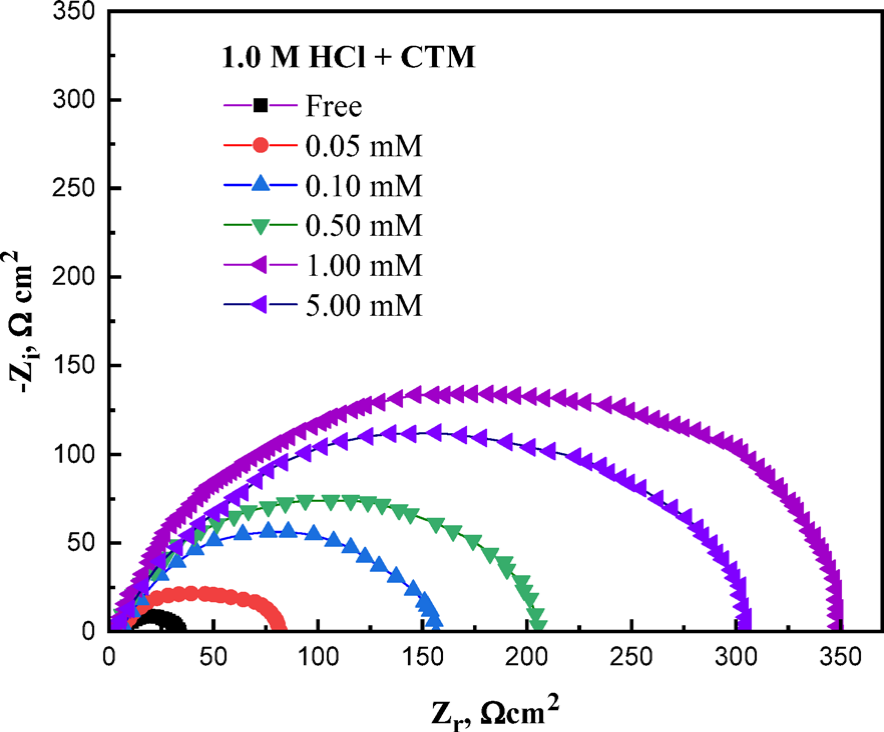
The Nyquist curves of the examined metal in 1.0 M hydrochloric acid solution, without and with various additions of CTM expired drug, at 25 °C.

**Fig. 5:**
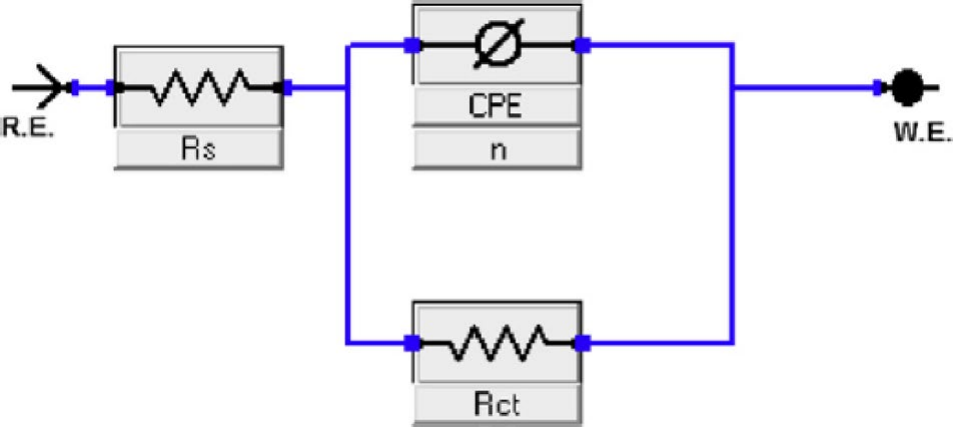
Equivalent electrical circuit used to fit the EIS data for the CTM inhibitor at 25 °C.

Electrochemical impedance spectroscopy (EIS) was employed as a rapid and non-destructive technique to investigate the interfacial behavior of carbon steel in 1.0 M HCl, both in the absence and presence of varying concentrations of expired CTM (Fig. 4). The impedance response of the steel surface changed significantly upon addition of the inhibitor, indicating modification of the metal/solution interface. The Nyquist plots were analyzed using the equivalent electrical circuit shown in Fig. 5^47^, which consists of the solution resistance (*R*_s_) in series with a parallel combination of charge transfer resistance (*R*_ct_) and a constant phase element (CPE). This circuit was selected because it effectively describes the electrochemical response of corroding metal surfaces where the corrosion process is predominantly controlled by charge transfer at the metal/solution interface.

The constant phase element (CPE) was used instead of an ideal double-layer capacitor to account for the non-ideal capacitive behavior commonly observed in corrosion systems. Such deviations arise from surface heterogeneity, roughness, uneven current distribution, and the presence of adsorbed inhibitor molecules on the metal surface. Consequently, the Nyquist plots exhibit depressed capacitive semicircles rather than ideal ones. The addition of CTM notably altered the capacitive loop characteristics compared to the blank solution, and the enlargement of these loops confirms the protective role of CTM against acid-induced corrosion, suggesting that the inhibition mechanism is predominantly governed by charge-transfer processes at the metal/solution interface^48,49^.

CTM molecules act as interfacial inhibitors by adsorbing onto the carbon steel surface and forming a protective barrier that limits direct interaction between the metal and the aggressive acidic medium^50^. Increasing CTM concentration enhances this protective effect, as evidenced by progressive changes in the capacitive loop features, indicating improved surface coverage and reduced metal dissolution.

**Fig. 6:**
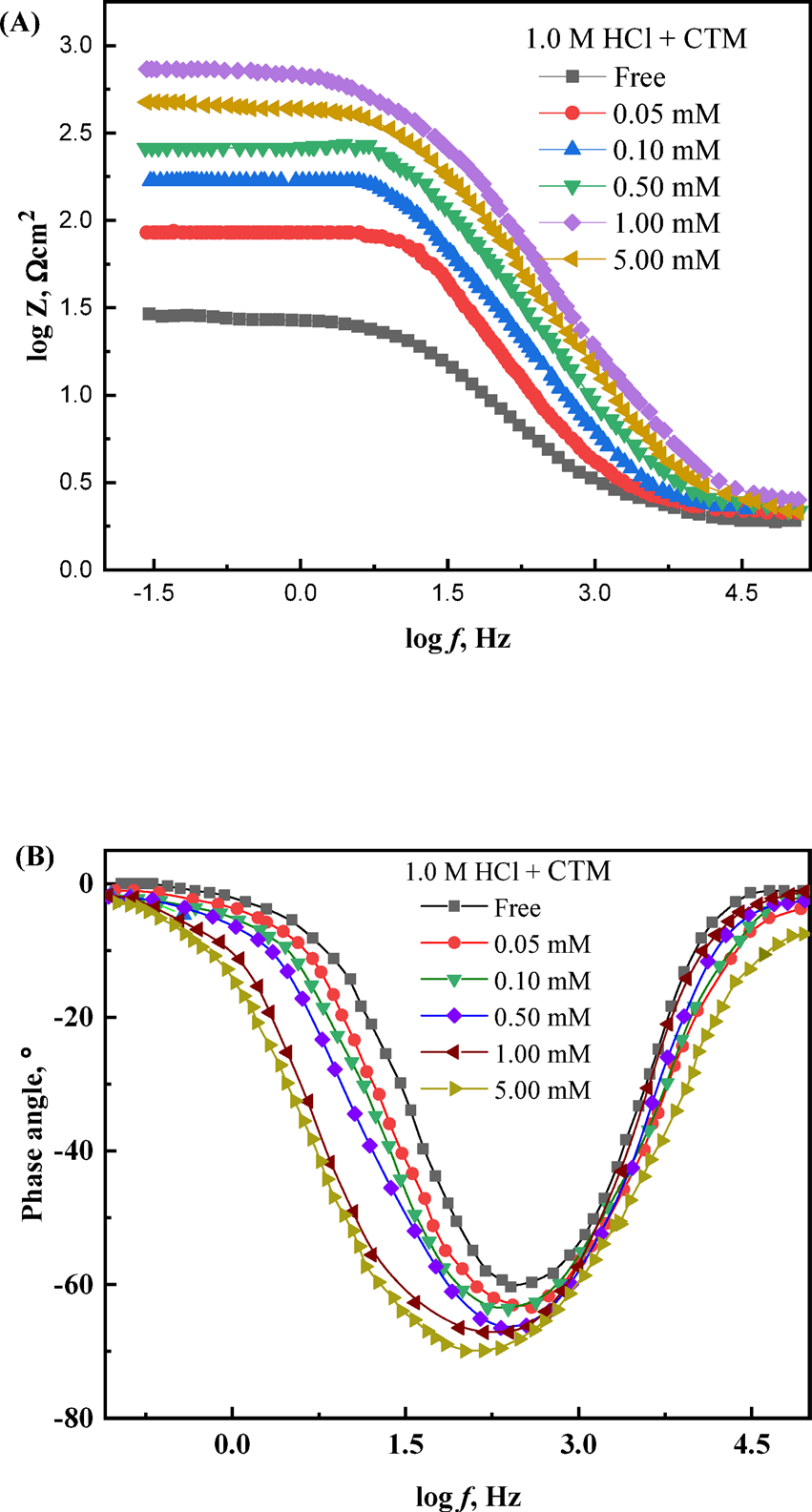
(**A**) Bode magnitude plots and (**B**) phase angle spectra for carbon steel in 1.0 M HCl in the absence and presence of various concentrations of CTM at 25 °C.

The inhibitive effect of expired CTM was further elucidated using Bode magnitude and phase angle plots (Fig. 6A and B). The Bode magnitude plots display a capacitive response at intermediate frequencies, while resistive behavior dominates at high and low frequencies. An increase in the maximum phase angle with higher CTM concentrations indicates formation of a protective surface film. Notably, the enhancement in inhibition efficiency (*η*_I_) correlates quantitatively with the observed EIS trends: *η*_I_ value increases from 50.2% for the lowest CTM concentration to 91.0% at the highest concentration, consistent with improved surface coverage^16,51^. Moreover, the phase angle shifts toward less positive values with increasing CTM dosage, confirming the development of an adsorbed inhibitive layer that reduces direct contact between the aggressive solution and the steel surface^16,52,53^.

Key electrochemical parameters extracted from impedance analysis, including charge transfer resistance (*R*_ct_), double-layer capacitance (*C*_dl_), and inhibition efficiency (*η*_I_), are summarized in Table 5. The progressive increase in *R*_ct_ with higher CTM concentrations reflects more effective adsorption of inhibitor molecules on the steel surface, which retards the metal dissolution process^16^. Conversely, the decrease in *C*_dl_ values with increasing CTM concentration is attributed to either an increase in the electrical double-layer thickness or a decrease in the local dielectric constant at the interface, further supporting the adsorption of CTM molecules and the consequent suppression of corrosion reactions^16,54^.

The n-values of the CPE obtained from fitting the impedance data (Table 5) range between 0.77 and 0.83, reflecting deviations from ideal capacitive behavior due to the inherent surface heterogeneity and roughness of the carbon steel surface. However, the slight increase in n-values with increasing CTM concentration indicates that the adsorbed inhibitor layer becomes more uniform and homogeneous. This behavior suggests that CTM adsorption smooths the electrochemical interface and improves surface coverage, resulting in the formation of a more compact and stable protective film. This interpretation is consistent with the observed increase in *R*_ct_ and the decrease in *C*_dl_ values, further confirming the effective inhibition performance of expired CTM.

**Table 5:** *EIS* parameters (*R*_ct_, *C*_dl_, and *η*_I_) for carbon steel in 1.0 M HCl in the absence and presence of different concentrations of expired CTM at 25 °C.

Conc., M	*R*_s_ Ω cm^2^	Q_dl_, Ω^−1^s^*n*^ cm^− 2^	*n*	Error of *n*	*R*_ct_, Ω cm^2^	C_dl,_ µF cm^− 2^	η_I_ %
0.00	2.54	0.0009771	0.77	1.22	25.4 *±* 0.35	280.4	-
5 × 10^− 5^	2.42	0.0002119	0.78	1.03	51.0 *±* 0.25	81.9	50.2
1 × 10^− 4^	2.59	0.0001803	0.75	1.18	74.1 *±* 0.21	72.8	65.7
5 × 10^− 4^	2.49	0.0001447	0.81	0.99	111.9 *±* 0.38	87.8	77.3
1 × 10^− 3^	2.81	0.0001108	0.79	0.87	182.7 *±* 0.42	76.7	86.1
5 × 10^− 3^	4.57	0.0000735	0.81	0.76	282.2 *±* 0.41	74.6	91.0

### Mechanism of adsorption

The inhibition of carbon steel corrosion by CTM in acidic media is primarily governed by the adsorption of inhibitor molecules onto the metal surface. This adsorption depends on the presence of multiple active sites in the CTM molecule and the solution temperature. The process follows a substitution mechanism, in which CTM molecules displace pre-adsorbed water molecules from the steel surface^55^:

11 $$\:{\mathrm{CTM}}_{\left(\mathrm{s}\mathrm{o}\mathrm{l}\right)}+n{\mathrm{H}}_{2}{\mathrm{O}}_{\left(\mathrm{a}\mathrm{d}\mathrm{s}\right)}\rightleftharpoons\:{\mathrm{CTM}}_{\left(\mathrm{a}\mathrm{d}\mathrm{s}\right)}+n{\mathrm{H}}_{2}{\mathrm{O}}_{\left(\mathrm{s}\mathrm{o}\mathrm{l}\right)}$$

where $\:n\:$represents the number of water molecules displaced per CTM molecule. Among various adsorption models tested (Freundlich, Temkin, Frumkin, and Langmuir), the Langmuir isotherm best describes the experimental data, indicating monolayer adsorption with negligible lateral interactions^56–58^:

12 $$\:\frac{C}{\theta\:}=\frac{1}{{K}_{ads}}+C$$

**Fig. 7:**
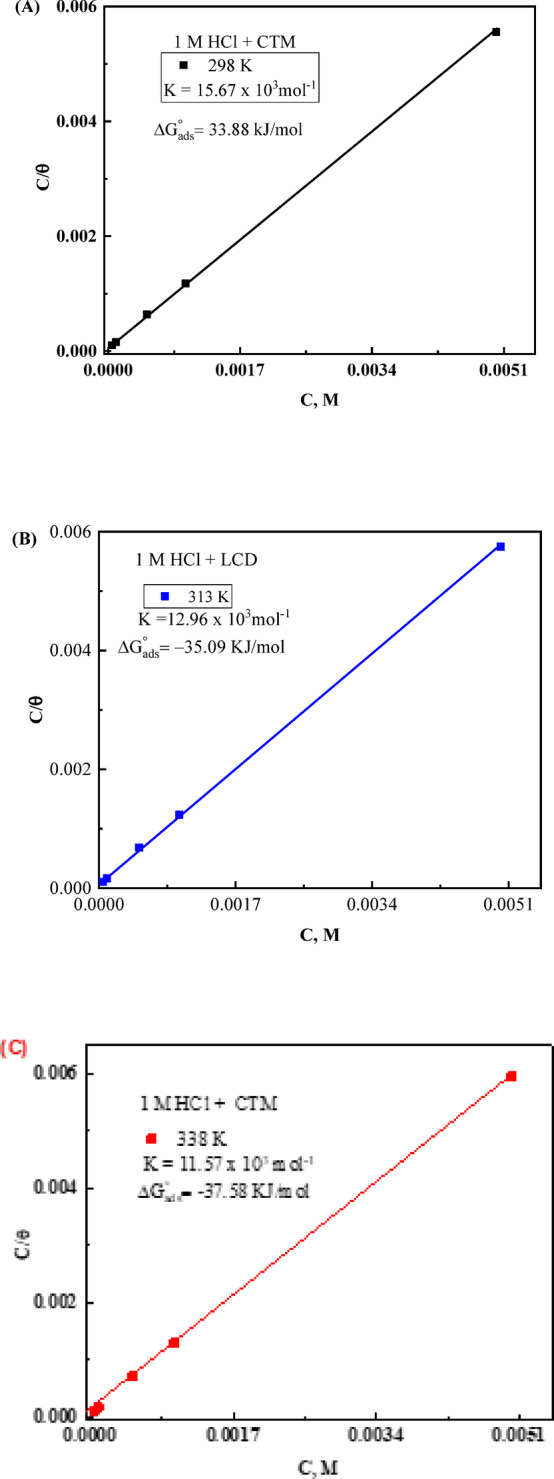
Langmuir isotherms (**A**,** B**, and **C**) for the CTM inhibitive molecules in 1 M HCl solution.

Here, *C* is the CTM concentration, *θ* is the surface coverage computed from gravimetric measurements, and *K*_ads_ is the adsorption–desorption equilibrium constant. The linear plots exhibited slopes near unity and high correlation coefficients (*R*² ≈ 1), confirming the Langmuir behavior. Figure 7(A-C) shows the linear relationship between *C*/θ and *C* at 25, 40, and 65 °C. The intercepts were used to calculate *K*_ads_ values, listed in Table 6. A clear decrease in *K*_ads_ with increasing temperature indicates partial desorption of CTM molecules from the carbon steel surface.

The standard free energy of adsorption (Δ*G*°_ads_) was calculated from the *K*_ads_ values using the following expression^59^:

13 $$\:{\Delta\:}{\mathrm{G}}_{\mathrm{a}\mathrm{d}\mathrm{s}}^{\circ\:}=-\mathrm{R}\mathrm{T}\left[\mathrm{l}\mathrm{n}{\mathrm{K}}_{\mathrm{a}\mathrm{d}\mathrm{s}}+4.0164\right]$$

The calculated ΔG°_ads_ values ranged between − 33.88 and − 37.58 kJ·mol⁻¹ (Table 6). The negative values confirm the spontaneous nature of the adsorption process^60,61^. Moreover, the magnitude of ΔG°_ads_ lying between − 20 and − 40 kJ mol⁻¹ suggests that CTM adsorption on carbon steel proceeds through a mixed physisorption–chemisorption mechanism^60^. The slight decrease in ΔG°_ads_ with increasing temperature further indicates that the inhibition process is endothermic in nature^46,61^. The obtained $\:{\Delta\:}{G}_{\mathrm{a}\mathrm{d}\mathrm{s}}^{\circ\:}$values ranged from − 33.88 to − 37.58 kJ mol⁻¹, confirming the spontaneous and strong adsorption of CTM on the carbon steel surface (Table 6)^60,61^. According to commonly accepted classifications, $\:{\Delta\:}{G}_{\mathrm{a}\mathrm{d}\mathrm{s}}^{\circ\:}$values around − 20 kJ mol⁻¹ are associated with electrostatic interactions (physisorption), whereas values more negative than − 40 kJ mol⁻¹ typically indicate chemisorption. Since the measured values fall within the intermediate range, the adsorption mechanism is best described as mixed physisorption–chemisorption, involving both electrostatic interactions and partial chemical bonding between the CTM molecules and the steel surface^60,61^.

The presence of heteroatoms such as nitrogen and oxygen in the CTM molecule, together with its bulky molecular structure, can promote donor–acceptor interactions with the vacant $\:d$-orbitals of iron atoms, thereby strengthening the adsorbed protective layer. The slight variation of $\:{\Delta\:}{G}_{ads}^{\circ\:}$with increasing temperature suggests a minor contribution of temperature-dependent interactions during the adsorption process.

This interpretation aligns with recent insights on complex adsorption mechanisms, highlighting that inhibitor adsorption often involves multiple interactions rather than a single chemisorption pathway, as discussed by Fan et al.^19^. Overall, the results indicate that CTM molecules form a stable, spontaneously adsorbed layer on the carbon steel surface, providing effective corrosion inhibition under acidic conditions.

**Fig. 8:**
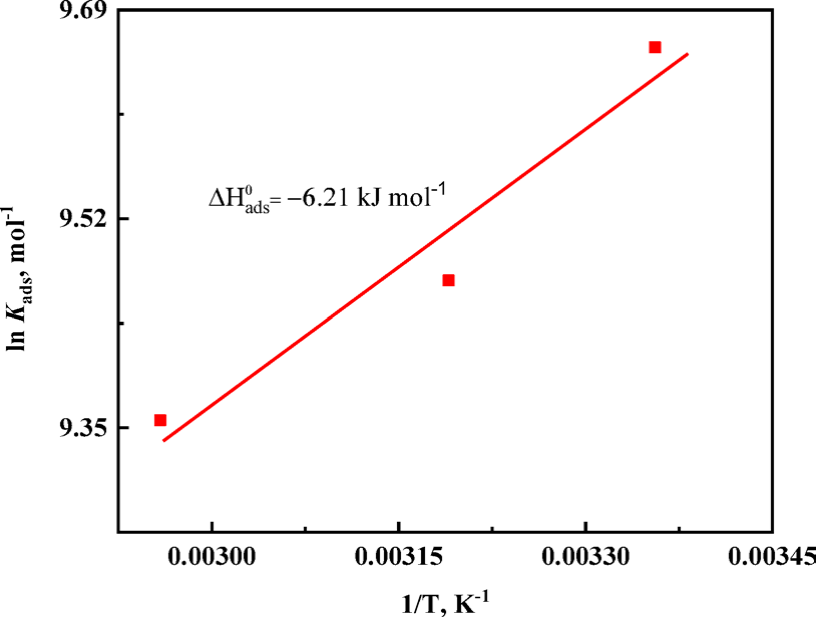
Variation of the ln *k* against T^− 1^ for the CTM inhibitor.

The thermodynamic parameters associated with CTM adsorption on carbon steel were evaluated using the Van’t Hoff and Gibbs free energy relationships. The Van’t Hoff equation^61^, expressed in Eq. (14), was used to calculate the standard enthalpy of adsorption. The obtained value, $\:{\Delta\:}{H}_{\mathrm{a}\mathrm{d}\mathrm{s}}^{\circ\:}=-6.21{\hspace{0.17em}}{\mathrm{kJ\:mol}}^{-1}$(Fig. 8; Table 6), indicates that the adsorption process is exothermic.

14 $$\:\mathrm{l}\mathrm{n}{K}_{\mathrm{a}\mathrm{d}\mathrm{s}}=-\frac{{\Delta\:}{H}_{\mathrm{a}\mathrm{d}\mathrm{s}}^{\circ\:}}{RT}+\mathrm{constant}$$

15 $$\:{{\Delta\:}G}_{\mathrm{a}\mathrm{d}\mathrm{s}}^{^\circ\:}=\:{{\Delta\:}H}_{\mathrm{a}\mathrm{d}\mathrm{s}}^{^\circ\:}\:\:-\:{\mathrm{T}{\Delta\:}S}_{\mathrm{a}\mathrm{d}\mathrm{s}}^{^\circ\:}$$

Further thermodynamic insight was obtained from the Van’t Hoff relationship. The standard enthalpy of adsorption ($\:{\Delta\:}{H}_{ads}^{\circ\:}$) was determined using the Van’t Hoff equation^61^:

16 $$\:\mathrm{l}\mathrm{n}{K}_{\mathrm{a}\mathrm{d}\mathrm{s}}=-\frac{{\Delta\:}{H}_{\mathrm{a}\mathrm{d}\mathrm{s}}^{\circ\:}}{RT}+\mathrm{constant}$$

The calculated value of $\:{\Delta\:}{H}_{ads}^{\circ\:}$(− 6.21 kJ mol⁻¹) (Fig. 8; Table 6) indicates that the adsorption process is exothermic and energetically favorable.

The entropy change of adsorption was obtained from the thermodynamic relation:

17 $$\:{\Delta\:}{G}_{ads}^{\circ\:}={\Delta\:}{H}_{ads}^{\circ\:}-T{\Delta\:}{S}_{ads}^{\circ\:}$$

The calculated entropy values ($\:{\Delta\:}{S}_{\mathrm{a}\mathrm{d}\mathrm{s}}^{\circ\:}$) were positive and nearly constant (92.3–92.9 J mol⁻¹ K⁻¹; Table 6). The positive entropy change suggests an increase in disorder at the metal–solution interface, which can be attributed to the displacement of pre-adsorbed water molecules by CTM molecules during the adsorption process.

**Table 6:** The thermodynamic adsorption parameters, *K*_ads_, ∆*G*^o^_ads_, ∆*H*^o^_ads_, and ∆*S*^o^_ads_, for the investigated CTM inhibitor, at various temperatures.

T, ℃	K_ads_ x10^− 3^, M^− 1^	∆G^o^_ads_, kJ mol^− 1^	∆H^o^_ads_, kJ mol⁻¹	∆S^o^_ads_, J mol^− 1^ K^− 1^
298	15.67	−33.88	−6.21	92.9
313	12.96	−35.09	−6.21	92.3
338	11.57	−37.58	−6.21	92.8

### Temperature study

**Fig. 9:**
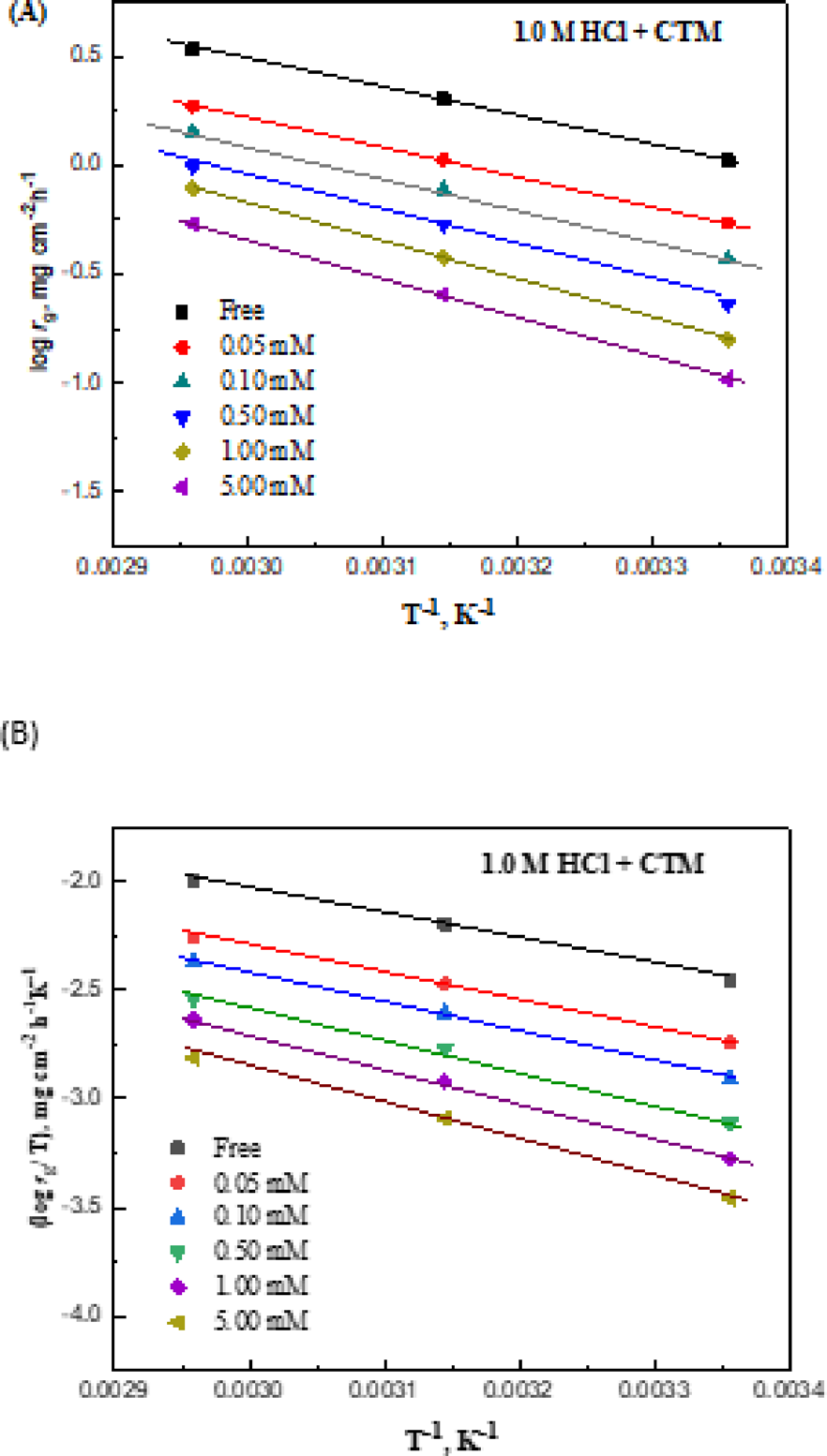
(**A**) Arrhenius plots and (**B**) transition-state plots for carbon steel in 1.0 M HCl in the absence and presence of different concentrations of the CTM inhibitor.

The activation energy (*E*_a_) for the corrosion of carbon steel was evaluated using the Arrhenius equation^59^:

18 $$\:\mathrm{l}\mathrm{o}\mathrm{g}({r}_{g})=\frac{-{E}_{a}}{2.303R}\cdot\:\frac{1}{T}+\mathrm{constant}$$

where R is the universal gas constant, and *r*_g_ is the corrosion rate obtained from the mass-loss method at different temperatures. Figure 9A shows the plot of *r*_g_ versus 1/T for both uninhibited and CTM-containing solutions. The linear relationships with high correlation coefficients confirm the applicability of the Arrhenius equation. The calculated *E*_a_ values (Table 7) increase in the presence of CTM, consistent with a decrease in inhibition efficiency, suggesting physisorption of CTM molecules on the steel surface^62–65^.

The enthalpy (ΔH*) and entropy (ΔS*) of activation were derived from the transition-state Eqs^66,67^:

19 $$\:\mathrm{l}\mathrm{o}\mathrm{g}\left(\frac{{r}_{g}}{T}\right)=\mathrm{l}\mathrm{o}\mathrm{g}\left(\frac{R}{{N}_{A}h}\right)+\frac{{\Delta\:}{S}^{*}}{2.303R}-\frac{{\Delta\:}{H}^{*}}{2.303RT}$$

where*h* and *N*_A_ are Planck’s and Avogadro’s constants, respectively. Figure 9B shows the log (*r*_g_/T) versus 1/T plots for both uninhibited and inhibited solutions. Linear fits were obtained, with slopes equal to −ΔH*/R. The computed ΔH* values (Table 7) are positive, indicating the endothermic nature of the transition state and supporting the reduction of the corrosion rate^45,68^.

The calculated ΔS* values are negative, ranging from − 171 J mol⁻¹ K⁻¹ in the blank HCl solution to − 172 to − 145 J·mol⁻¹·K⁻¹ with increasing CTM concentration (Table 7). The negative entropy reflects the stability of the adsorbed inhibitor film on the metal surface, while the gradual increase in ΔS* with higher CTM concentrations suggests that the activated complex in the rate-determining step involves an associative process rather than dissociation^38,69–73^.

**Table 7:** The thermodynamic activation values of *E*_a_, ∆H*_ads_, and ∆S*_ads_ for the CTM compound.

Conc. of Cp (mM)	E_a_ (kJ.mol^− 1^)	∆H*_ads_ (kJ.mol^− 1^)	∆S*_ads_ (J.mol^− 1^.K^− 1^)
Free	24.55	21.90	−171
0.05	25.89	23.28	−172
0.10	28.07	26.29	−165
0.50	30.54	27.92	−163
1.00	33.53	30.90	−147
5.00	34.46	31.27	−145

### Surface examination

**Fig. 10:**
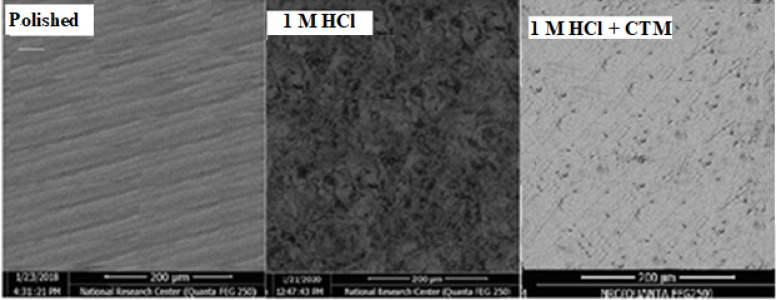
SEM micrographs of the examined steel without immersion (**A**), and with immersion in a blank free (**B**), and containing 0.005 M CTM compound (**C**), successively.

**Fig. 11:**
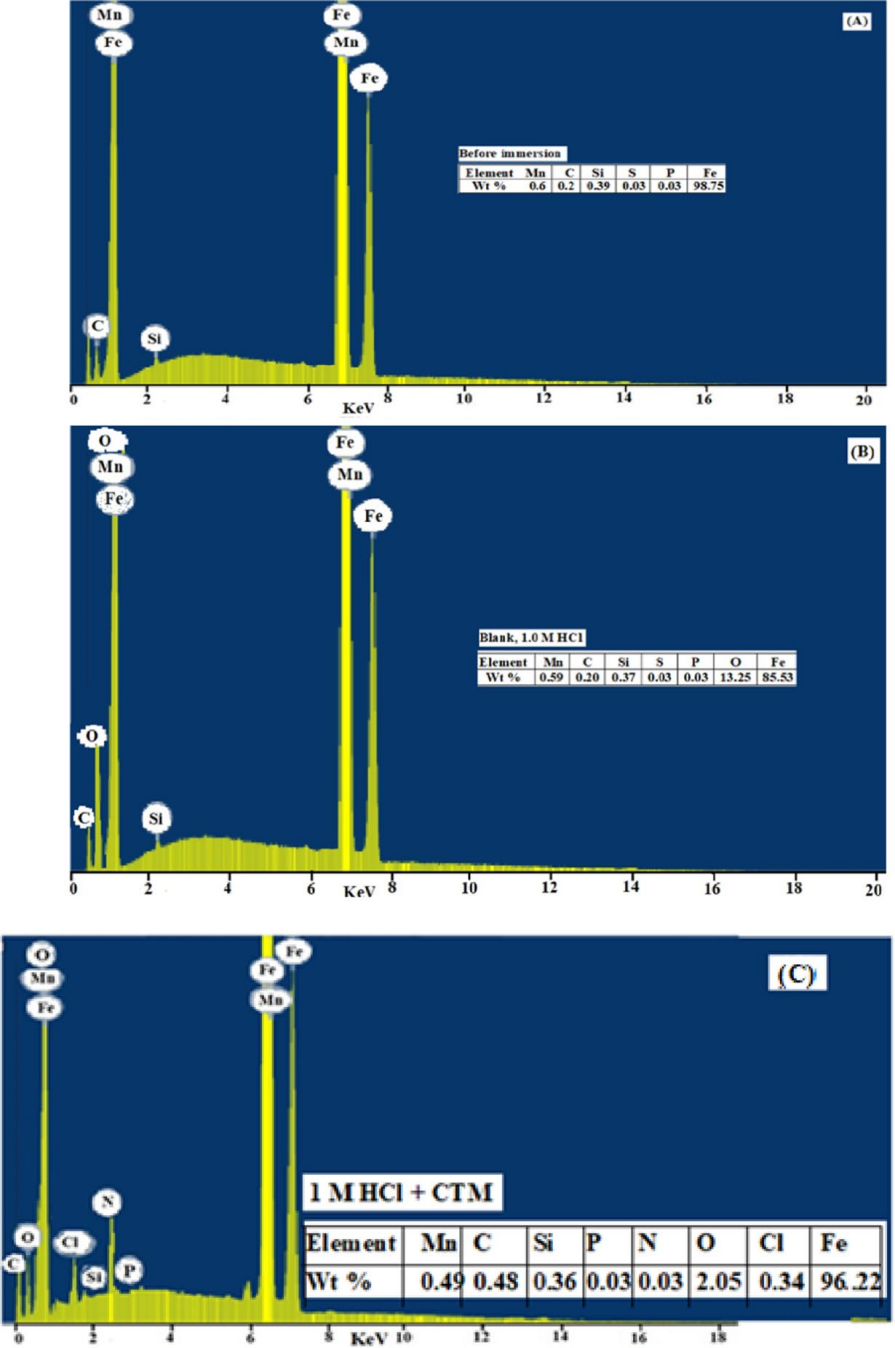
EDX spectra of carbon steel surface: (**A**) before immersion, (**B**) after immersion in blank 1.0 M HCl solution, and (**C**) after immersion in 1.0 M HCl containing 5 mM CTM.

In contrast, Fig. 10B illustrates the morphology of the carbon steel surface after immersion in a dilute HCl solution for 8 h. The surface appears severely damaged and covered with corrosion products due to the aggressive action of the acidic medium. The EDS spectrum of this sample (Fig. 11B) indicates a marked decrease in the iron content (Fe = 85.53%), confirming significant metal dissolution in the absence of the inhibitor.

Figure 10C presents the SEM micrograph of the steel surface after 8 h immersion in dilute HCl containing 5 mM CTM. Compared to the uninhibited sample, the surface appears much less damaged, with fewer corrosion features. This improvement can be attributed to the formation of a protective adsorbed film of CTM molecules on the metal surface. The corresponding EDX analysis (11 C) shows a higher Fe percentage (Fe = 96.22%) than that obtained in the inhibitor-free solution, supporting the presence of an adsorbed CTM layer that limits iron dissolution.

Overall, the SEM and EDX results clearly demonstrate that CTM effectively reduces carbon steel corrosion in HCl solution through adsorption and protective film formation, thereby minimizing iron loss into the acidic medium.

## Conclusion

- Various experimental techniques confirmed that the CTM drug acts as a mixed-type inhibitor for carbon steel corrosion in 1.0 M HCl solution.
- The inhibition efficiency increases with increasing CTM concentration and decreases with rising temperature.
- The inhibition mechanism is governed by the adsorption of CTM molecules on the steel surface, following the Langmuir adsorption isotherm.
- The decrease in surface coverage (θ) and inhibition efficiency with increasing temperature suggests partial desorption of inhibitor molecules, indicating a contribution of physical adsorption.
- The relatively high *K*_ads_ values and the negative Δ*G*^o^_ads_ values indicate strong adsorption of CTM molecules onto the steel surface and confirm the spontaneous nature of the adsorption process.
- The Δ*G*^o^_ads_ values (− 33.88 to − 37.58) together with ∆*H*^o^_ads_ (− 6.21 kJ mol⁻¹) suggest that the adsorption mechanism involves both physical and chemical interactions, with a dominant chemisorption character.

## Data Availability

The datasets used or analyzed in the current study are included in the text.
